# Nitric oxide and L-type calcium channel influences the changes in arterial blood pressure and heart rate induced by central angiotesin II

**DOI:** 10.1186/1744-9081-4-22

**Published:** 2008-05-24

**Authors:** Wilson A Saad, Ismael FMS Guarda, Luiz AA Camargo, Talmir AFB Santos

**Affiliations:** 1 Basic Institute of Biosciences-University of Taubaté, Taubaté, Brazil; 2 Department of Exact and Natural Science Araraquara University Center, Araraquara, Brazil; 3 Department of Physiology and Pathology School of Dentistry, Paulista State University, Araraquara, Brasil; 4 Department of Anesthesiology Clinic Hospital State of São Paulo ,São Paulo, Brazil; 5 Department of Physiology, Federal University of São Carlos, Brazil

## Abstract

We study the voltage dependent calcium channels and nitric oxide involvement in angiotensin II-induced pressor effect. The antipressor action of L-Type calcium channel antagonist, nifedipine, has been studied when it was injected into the third ventricle prior to angiotensin II. The influence of nitric oxide on nifedipine antipressor action has also been studied by utilizing N^W^-nitro-L-arginine methyl ester (LNAME) (40 μg/0.2 μl) a nitric oxide synthase inhibitor and L-arginine (20 μg/0.2 μl), a nitric oxide donor agent. Adult male Holtzman rats weighting 200–250 g, with cannulae implanted into the third ventricle were injected with angiotensin II. Angiotensin II produced an elevation in mean arterial pressure and a decreased in heart rate. Such effects were potentiated by the prior injection of LNAME. L-arginine and nifedipine blocked the effects of angiotensin II. These data showed the involvement of L-Type calcium channel and a free radical gas nitric oxide in the central control of angiotensin II-induced pressor effect. This suggested that L-Type calcium channel of the circunventricular structures of central nervous system participated in both short and long term neuronal actions of ANG II with the influence of nitrergic system.

## Background

Nitric oxide plays an important role in the hydromineral and cardiovascular regulation and influence several central angiotensin physiological parameters [1,2]. LNAME increases blood pressure which is at least in part salt sensitive [3]. A major factor that detemined a neuronal calcium-dependent signal is the opening of permeability pathways for calcium in the cell membrane [4]. However, the interaction between nifedipine (L-Type calcium channel blocked agent) and nitrergic system of the circumventricular third ventricle structures of the central nervous system on the angiotensin II cardiovascular regulation has not been demonstrated.

The subfornical organ is an important circumventricular structure of the central nervous system that participated in the regulation of body fluid homeostasis [5-7]. LNAME significantly increased the discharge of neurons of the subfornical organ showing the importance of nitric oxide in the electrical activity of this structure [8]. FK 409 (a nitric oxide donor agent) injected into median preoptic nucleus of conscious rats decreased mean arterial pressure [9].

Treatment of neonatal rats with monosodium glutamate induced a substantial reduction in the volume of the subfornical organ and in the number of its nitrergic cells with regards to control animals [10]. These findings suggested that the subfornical organ could be implicated in some physiological functions such as salivary secretion and cardiovascular alterations observed in monosodium glutamate-treated rats.

The objective of this study was to determined the role of voltage-sensitive calcium channels in angiotensin II-induced pressor response when it was injected into the third ventricle of conscious rats. We also studied the influence of a nitric oxide on the nifedipine effect.

## Methods

The Medical Ethics Committee of the Universidade Estadual Paulista UNESP approved all protocols in this study.

Male Holtzman rats weighing 250–300 g were anesthetized with ketamine (80 mg/Kg of body weight) plus xylazine (7 mg/Kg of body weight). A stainless steel cannula with 10 and 12-mm long and 0.7-mm OD was implanted into the 3^rd ^V according to the coordinates of Paxinos and Watson atlas rat brain [11].

After the animals recovery from brain surgery (5 days) PE-10 polyethylene tubing connected to PE-50 tubing was inserted into the abdominal aorta through the femoral artery. Direct mean arterial pressure and heart rate was record in unaesthetized and unrestrained rats. The animals were removed from their home cages and placed in test cages, without access to food or water. The previously implanted catheter was connected to a Statham (P23 Db) pressure transducer (Statham-Gould, Valley View, OH) coupled to a multi channel recorded (PowerLab Multirecord). This program permits the acquisition of cardiovascular data by computer. In these experiments the rats were chosen at random. Each animal was used no more than tree times.

The drugs were injected into the third ventricle by using a Hamilton micro syringe (5 μl) connected by a PE-10 polyethylene tubing (25 cm) to a needle (0.3 mm o. d.), which was introduced into the brain through the cannula previously fixed to the animals' head.

Mean arterial pressure and heart rate were record in conscious rats in a test cage, without access to food or water.

The results are reported as mean ± S.E.M. The Analysis of variance and Newman-Keuls post-hoc test were used to determine the significance. The values were considered statistically significant with 5% level (*p *< 0.05).

## Results

**Figure 1:**
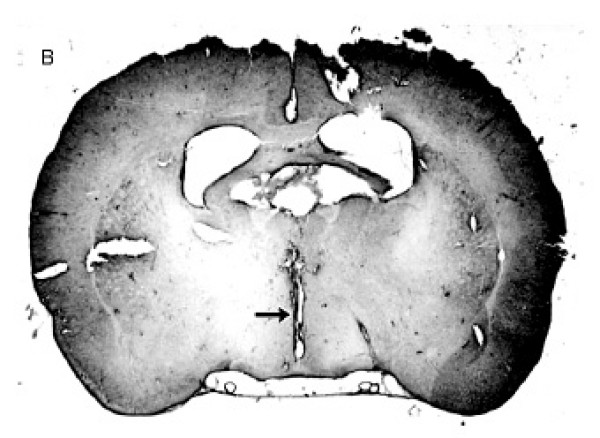
Photomicrograph of the brain showing the place reached by the cannula into the 3^rd ^V (arrow).

At the end of the experiments, the rats were anesthetized with ether and perfused with saline and buffered formalin. The brains were removed, fixed in 10% formalin, frozen to -25°C and cut into 20–30 μm coronal sections. Only animals in which the injection was placed into the third ventricle were used in this study (figure 1).

### Effects of nifedipine and L-arginine on the mean arterial pressure and heart rate induced by the injection of angiotensin II into the third ventricle Figure 2, 3

**Figure 2:**
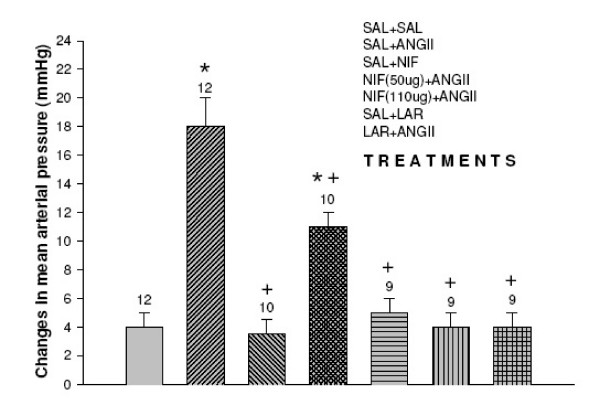
Effect of nifedipine and L-arginine on mean arterial pressure induced by the injection of angiotensin II into the third ventricle. Number of animals at the top of each column. Data are means ± S.E.M. *p < 0.05 vs.saline+saline, ^+^*P *< 0.05 vs. saline+angiotensin II (Neuman-Keuls post-hoc test).

**Figure 3:**
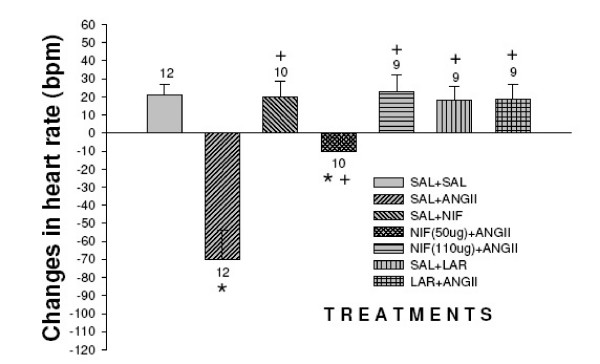
Effect of nifedipine and L-arginine on heart rate induced by the injection of angiotensin II into the third ventricle. Number of animals at the top of each column. Data are means ± S.E.M. *p < 0.05 vs.saline+saline, ^+^*P *< 0.05 vs. saline+angiotensin II (Neuman-Keuls post-hoc test).

Microinjections of angiotensin II into the third ventricle induced increase in means arterial pressure compared to control (Δ18 ± 2 vs. control Δ4 ± 1 mmHg *p *< 0.05) and decreased heart rate compared to control Δ-70 ± 16 vs. 21 ± 9 bpm *p *< 0.05). The microinjection of saline+nifedipine into the third ventricle caused no change in the mean arterial pressure (Δ3.5 ± 1 mmHg) and heart rate (Δ20 ± 9 bpm). Nifedipine 50 μg injected into the third ventricle followed by angiotensin II decreased the pressor effect (Δ11 ± 1 mmHg *p *< 0.01) with a decreased in heart rate Δ-10 ± 2 bpm *p *< 0.01). Nifedipine 100 μg injected into the third ventricle followed by angiotensin II blocked angiotensin II-pressor effect (5 ± 1 mmHg *p *< 0.01) and no changes in heart rate was observed (Δ23 ± 9 bpm). The injection of L-arginine also blocked the pressor effect of angiotensin II (Δ4 ± 1 mmHg *p *< 0.01) no alterations in heart rate was observed (Δ19 ± 8 bpm). No alterations in mean arterial pressure and in heart rat was observed when L-arginine was injected alone into the third ventricle (*F *_(6,64) _= 63.4, *p *< 0.01) and (*F *_(6,64) _= 63.4, *p *< 0.01) respectively.

### Effects of nifedipine and LNAME on the mean arterial pressure and heart rate induced by the injection of angiotensin II into the third ventricle Figure 4, 5

**Figure 4:**
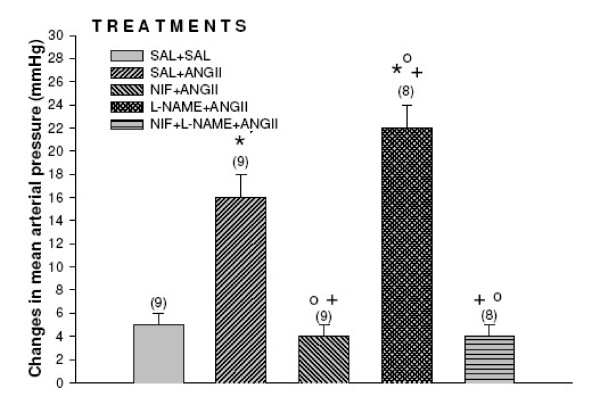
Effects of nifedipine and LNAME on the mean arterial pressure induced by the injection of angiotensin II into the third ventricle. Number of animals at the top of each column. Data are means ± S.E.M. *P < 0.05 vs. saline+saline, ^+^*P *< 0.05 vs. saline+angiotensin II and ^O^P < 0.05 vs. LNAME+angiotensin II (Neuman-Keuls post-hoc test).

**Figure 5:**
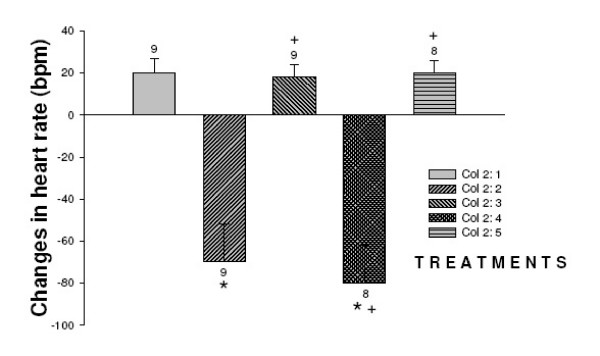
Effects of nifedipine and LNAME on heart rate induced by the injection of angiotensin II into the third ventricle. Number of animals at the top of each column. Data are means ± S.E.M. *P < 0.05 vs. saline+saline, ^+^*P *< 0.05 vs. saline+angiotensin II and ^O^P < 0.05 vs. LNAME+angiotensin II (Neuman-Keuls post-hoc test).

Angiotensin II injected into the third ventricle increased mean arterial pressure and decreased heart rate. Nifedipine injected prior to angiotensin II decreased mean arterial pressure without changes in heart rate (*F *_(4,38) _= 81.3 *P *< 0.01) and *F *_(4,38) _= 78.3 *P *< 0.01 respectively. The increased of mean arterial pressure induced by angiotensin II (Δ17 ± 1 mmHg) was potentiated by L-NAME (25 ± 2 mmHg *p *< 0.01) with decreased in heart rate (Δ-60 ± 6 vs. -80 ± 10 bpm *p *< 0.05). Rats injected with nifedipine 100 μg prior to L-NAME followed by angiotensin II decreased the mean arterial presure (7 ± 2 mmHg *p *< 0.01) no alterations in heart rate was ovserved (20 ± 1 bpm) (*F *_(4,38) _= 81.3 *P *< 0.01) and *F *_(4,38) _= 78.3 *P *< 0.01 respectively.

## Discussion

In these experiments microinjections of angiotensin II into the third ventricle increased mean arterial pressure and decreased heart rate. which were blocked by nifedipine and potentiated by L-NAME. The microinjection of L-arginine into third ventricle decreased the pressor effect of angiotensin II no alterations in heart rate was opvserved. The injection of nifedipine combined with L-NAME, which were injected prior to angiotensin II into the third ventricle, blocked the potentiation effect of L-NAME. It has been demonstrated that injection of L-NAME into the median proptic area increased the mean arterial pressure [12].

The treatment with L-NAME increased arterial blood pressure, which is at least in part salt sensitive. The action of L-NAME also may be due to a local vasoconstriction. Furthermore, the salt-sensitive component appears to be angiotensin II-dependent, as it was associated with increase of plasma angiotensin II levels and could be reversed by the treatment with angiotensin II receptor antagonist [3].

These data, suggest that structures surrounding cerebral ventricles may release angiotensin II which acts as a neurotransmitter resulted in postsynaptic effects, which in turn influenced blood pressure and heart rate control. Angiotensinergic neural pathways and calcium channels are important in neural function and may have important homeostatic roles, particularly related to cardiovascular function by involved nitric oxide. It has been demonstrated that niric oxide antagonized the vasoconstrictive and pro-atherosclerotic effects of angiotensin II whereas angiotensin II decreased nitric oxide bioavailability by promoting oxidative stress.

By the results of the present study we can suggested that nifedipine may have interfered with calcium influx in the presynaptic terminals, where L-type calcium channels play important roles in modulated presynaptic neurotransmitter release. It may also have altered Ca^2+^-dependent signal events in postsynaptic neurons since previous studies demonstrated the permissive effects of voltage sensitive calcium channels on monosodium glutamate-treated rats receptor-mediated calcium influx. In most neurons of the central nervous system, there are at least two major classes of calcium channel: voltage sensitive and receptor-operated calcium channels. Ours results are strongly supported by the results of others that demonstrated the hypertensive effect of angiotensin II significantly enhanced by prior microinjection of LNAME into paraventricular nucleus this showed that the effect of angiotensin II in arterial blood pressure interact with nitric oxide [13-16].

## Conclusion

The main find of these experiments is that nifedipine with or without L-NAME blocked the effect of angiotensin II on arterial blood pressure and heart rate. We can explained that the pressor effect of angiotensin II injected into third ventricle acted into the circumventricular structures and utilized L-Type calcium channel to exerted it effects. The nitric oxide also participated in angiotensin II effect. The influence of L-Type calcium channel and nitric oxide utilizing cGMP pathways on angiotensin II pressor effect explained these results. Finally we demonstrated that angiotensin II utilized the calcium channel and nitric oxide to exerced the central regulation of arterial blood and heart rate. These results provide support to future studies that involved specific circumventricular structures.

## List of abreviations

SAL: saline; ANGII: angiotensin II; LAR: L-arginine; NIF: nifedipine; L-NAME: L-NAME

## Competing interests

The authors declare that they have no competing interests.

## Authors' contributions

WAS made substantial contributions to conception and design, or acquisition of data

IFMSG analyzed the data and have been involved in drafting the manuscript

LAAC revised and made critically for important intellectual content

TAFBS made the figures and performed the statistical analysis
